# Nutritional and Acquired Deficiencies in Inositol Bioavailability. Correlations with Metabolic Disorders

**DOI:** 10.3390/ijms18102187

**Published:** 2017-10-20

**Authors:** Simona Dinicola, Mirko Minini, Vittorio Unfer, Roberto Verna, Alessandra Cucina, Mariano Bizzarri

**Affiliations:** 1 Department of Experimental Medicine, Systems Biology Group, Sapienza University of Rome, viale Regina Elena 324, 00161 Rome, Italy; simona.dinicola@uniroma1.it (S.D.); mik92x@hotmail.it (M.M.); roberto.verna@uniroma1.it (R.V.); 2 Department of Surgery “Pietro Valdoni”, Sapienza University of Rome, Via Antonio Scarpa 14, 00161 Rome, Italy; alessandra.cucina@uniroma1.it; 3 Department of Medical Sciences, IPUS-Institute of Higher Education, 5250 Chiasso, Switzerland; vunfer@gmail.com; 4 Policlinico Umberto I, viale del Policlinico 155, 00161 Rome, Italy

**Keywords:** myo-Inositol, phytate (InsP6), diabetes, myo-inositol oxygenase (MIOX), diabetic nephropathy, cancer, inositol hexakisphosphate kinase (IP6K1), phosphatidic acid, Inositol-3-Phosphate Synthase 1 (ISYNA1)

## Abstract

Communities eating a western-like diet, rich in fat, sugar and significantly deprived of fibers, share a relevant increased risk of both metabolic and cancerous diseases. Even more remarkable is that a low-fiber diet lacks some key components—as phytates and inositols—for which a mechanistic link has been clearly established in the pathogenesis of both cancer and metabolic illness. Reduced bioavailability of inositol in living organisms could arise from reduced food supply or from metabolism deregulation. Inositol deregulation has been found in a number of conditions mechanistically and epidemiologically associated to high-glucose diets or altered glucose metabolism. Indeed, high glucose levels hinder inositol availability by increasing its degradation and by inhibiting both myo-Ins biosynthesis and absorption. These underappreciated mechanisms may likely account for acquired, metabolic deficiency in inositol bioavailability.

## 1. Introduction. Health Protection from Whole-Grain Cereals: Key Components beyond Fiber

The protective role of a diet with a high content of fibers against the development of chronic diseases—including obesity, diabetes, polycystic ovary syndrome (PCOS), metabolic syndrome, cardiovascular diseases, and cancer—is well established [1,2,3,4]. This effect is mostly due to the presence of whole-grain cereal products. Noticeably, whole-grain cereal consumption has also been shown to be protective against mortality for inflammatory-related diseases [5]. Conversely, consumption of high-refined diet (namely for alimentary regimens that are mostly based on pasta, rice, and bread) has been associated with increased risk for cancer [6]. Additionally, a high-fiber diet comprises a huge number of phytochemicals, including polyphenols, bioflavonoids, vitamins, oligo elements, betaine, choline, phytosterols, and melatonin. Each of these compounds displays a wide range of pharmacological activities of potential interest. Indeed, several physiological functions and acknowledged health benefits have been documented by both in vitro and in vivo studies [7]. Therefore, it is practically impossible to assign to only few molecular components the whole body of recorded biological effects. Conversely, it is likely that such effects could be explained by the synergistic cooperation among those compounds, acting as a whole, like a “poly-pharmacological network”, able to influence several targets at the same time [8].

While a few different hypotheses have been proposed to explain the protective role exerted by diets with high-fiber content [9], some early reports pointed out the presence of inositol(s) as causative protective agents. Such studies demonstrated that only fibers with high phytate content, such as cereals and legumes, show negative correlation with colon cancer, indicating that phytate, and not fiber, suppressed colon carcinogenesis [10].

Moreover, it was showed that phytic acid exerts some effects originally attributed to fibers. Phytate increases the weights of the cecum and cecal digesta and reduces pH of cecal digesta [11]. In addition, phytate improves the composition of cecal organic acids, microflora, and mucins, and decreases the levels of serum proinflammatory cytokines in rats fed a high-fat, mineral-sufficient diet [12].

Indeed, myo-Inositol (myo-Ins) and its phosphate derivatives (namely, Inositol-hexakisphosphate, InsP6, or phytic acid) have been demonstrated to exert a plethora of valuable health effects—including anti-diabetic, anti-oxidant, anti-inflammatory, and anticancer effects [13]—thus gaining momentum in recent decades [14]. For instance, in a very preliminary study, F344 rats were injected with azoxymethane to induce colon tumors. Colon cancer development was heralded by the appearance of aberrant crypts in colon epithelium. In the absence of surgical excision, these inflammatory-like lesions progressed towards full neoplastic transformation. Adding InsP6 to the drinking water significantly reduced the number and depth of the colonic crypts, as well as the incidence of colon tumors (83% in controls versus 25% in rats treated with InsP6) [15]. These preliminary results have been further strengthened by additional investigations, which evidenced that both InsP6 and Inositol play some appreciable anticancer effects in a number of in vitro and in vivo studies (reviewed in [16]).

The inositol-dependent anticancer effects paved the way for discovering further properties of myo-Ins and its phosphate derivatives. Hence, in recent decades many attempts have been conducted to deepen the understanding of inositol involvement in different physiological and pathological conditions, including fertility [17,18], regenerative processes [19], oogenesis [20] and sperm function [21], glucose [22] and fat metabolism [23], morphogenesis [24,25], neurological disorders [26], and respiratory function in newborn [27].

Those studies highlight the relevance of the contribution of inositols in the development of several diseases, as well as how critical may be their uses as adjuvant treatment.

Nevertheless, few investigations specifically aimed at ascertaining how and why a critical deficiency in inositol(s) could happen and how that condition could favor the onset of pathological diseases have hitherto been published. The present survey aims precisely to contribute to filling that gap.

## 2. Inositol and Phytic Acid

Inositol (myo-Ins) belongs to the cyclitols family, which comprises nine isomers of the hexahydroxycyclohexane [28]. Myo-Ins is found in human diet in its free form, as inositol-containing phospholipid, and as InsP6. While inositol from animal sources is mostly represented in its free form or as inositol-containing phospholipid (phosphatidylinositol, PI) [29], in plant foodstuffs inositol is preferentially present as InsP6 [30]. As inositol phosphates are water soluble, in the daily human diet, the main sources of phytate are cereals and legumes, including oil, seeds and nuts.

Phytic acid is also the main source of phosphorus in both plants and mammals. Bacterial phytases and phosphatases—homologous of the mammalian InsP6 phosphatase—are responsible for digesting dietary InsP6, releasing free myo-Ins, orthophosphate, and intermediary inositol-phosphate derivatives (mono-, di-, tri-, tetra-, and penta-phosphate esters of inositol [31,32].

Inositol is an essential factor enabling growth of both normal and cancerous tissues in cultures [33], and for that reason, it has been considered for a while as an essential nutrient, belonging to the vitamin B family [34]. However, this assumption is no longer tenable as it was further observed that the human body (namely liver and kidney) [35] could produce up to 4 g/day on inositol, while a mixed western diet provides humans with roughly 1 g/day of myo-Ins [36]. Given that myo-Ins can be obtained through biosynthetic mechanisms as well as from dietary source, one would surmise that inositol deficiency is unlikely to happen. However, alterations in myo-Ins biosynthesis or reduced food supply have already been noticed. The resulting inositol deficiency may thus contribute to the development of numerous diseases.

Absorption of myo-Ins, PI, or inositol-phosphate derivatives (including InsP6) occurs in the gut. Phosphatidyl-inositol is hydrolyzed by a pancreatic phospholipase-A, and the resulting lyso-phosphatidyl-Inositol enters the intestinal cell where it is re-acetylated or further hydrolyzed with the formation of glycerylphosphorylinositol [32,34].

Studies in humans showed that 37–66% of dietary phytate is degraded during digestion in the stomach and small intestine when the diet is rich in plant food phytases [37]. Yet, as processing of food is likely to inactivate most of nutritional-derived phytases, in humans the main phytate hydrolysis occurs in the large intestine by means of microbial phytases [38].

Given that the inositol moiety very small in respect to phosphate groups, phytic acid has an extremely high negative charge density. Hence, it has been assumed that InsP6 cannot cross the lipid bilayer of plasma membranes. Given that adequate carriers have not yet been detected in the gut, for a while the gastro-intestinal absorption of phytate was considered rather improbable. However, besides some recent controversies [39,40], compelling evidence of a direct absorption of InsP6 from gastrointestinal cells has been provided since the 1980s [41], and received later firm confirmation [42], especially when InsP6 was directly measured through quantitative methods based on LC-MS bioanalytical procedures [43].

Several studies have demonstrated that the solubility of inositol phosphates in the stomach chyme (and hence their relative absorbability) critically depends to the degree of phosphorylation. The greater the degree of phosphorylation of inositol, the lower its solubility in the intestinal chyme and vice versa [44].

Ultimately, phytic acid is absorbed after oral ingestion and distributed throughout the body [45,46], reaching a peak in plasma concentration after 4 hours, even if the overall amount of InsP6 absorbed is only a minimal percentage of the administered dose. InsP6 in excess is excreted as such in the urine, even if there is a maximum excretion level that cannot be exceeded by ingesting higher amounts of InsP6 [47]. However, much of the ingested InsP6 is absorbed after hydrolyzation to myo-Ins (or into one of its phosphate derivatives), even if the majority of the InsP6 found in organs and tissues has a dietary origin and is not a consequence of endogenous synthesis [48].

For a long time, InsP6 was considered as an antinutrient due to its capacity to form insoluble salts with cations such as zinc, magnesium, potassium, iron, and copper in vitro. These findings paved the way for hypothesizing a decrease in the bioavailability of these elements in vivo, upon a diet rich in InsP6 [49]. Since then, besides some residual controversies [50,51,52], numerous studies clarified that the supposed “antinutritional” effect emerges only when large quantities of InsP6 are consumed in combination with an unbalanced, oligo elements-poor diet [53,54]. Indeed, a consumption of 1–2 g InsP6—as occurs in a standard western diet—does not significantly affect the mineral status in humans [55].

On the other hand, it has been surmised that the same chelating capabilities of InsP6 could provide a paradoxical advantage in preventing renal stone development (by forming insoluble complexes with calcium ions) [56,57,58], as well as microcalcification deposits in breast parenchyma, given that InsP6 is a strong inhibitor of calcium oxalate crystal formation in vitro [59]. Ultimately, it is tempting to speculate if InsP6 (or other inositol-phosphate derivatives) could also prevent to some extent Nickel-based diseases [60], given that it has already been proven that inositol-phosphates can chelate Nickel ions with high affinity [61,62].

On the contrary, myo-Ins do not form stable complexes with oligo elements in the gastrointestinal tract and it accumulates into intestinal cells in a Na^+^-dependent manner [63]. Glucose exerts a competitive inhibition on cellular myo-Ins uptake in both the gut and the kidney—thus hampering the tubular reabsorption of inositol—while Ca^2+^ ions do not seem to sensibly influence these processes [41]. Lipid composition of a meal may appreciably influence myo-Ins intestinal uptake, and pharmacokinetic studies with different myo-Ins formulations showed that oral myo-Ins availability is increased when administered in soft gel capsules [64].

## 3. Inositol Deficiency

Inositol deficiency registered some interest during recent decades, mostly in an attempt to explain the therapeutic mechanism of lithium, credited to act by inducing a depletion of cellular inositol [65].

However, inositol deficiency may arise through a plethora of different mechanisms—including reduced food-dependent intake, increased catabolism, and excretion, decreased biosynthesis, inhibition of intestinal and cellular uptake—and can significantly affect several human pathological conditions [66]. In addition, despite myo-Ins deficiency being ascertained in different animal species [67], requirements for dietary inositol in man have still not been explicitly assessed. In addition, reliable estimation can be difficult because dietary necessities may be higher depending on the person’s age, the long-term use of antibiotics, or the regular consumption of a high amount of coffee [68] or other unknown diet components.

Furthermore, a major source of variation depends on the myo-Ins and InsP6 actual content of available foods. For most cereals and fruits, fluctuations in inositol content may account for differences of one or two orders of magnitude. This variance does not only reflect the great number of botanical varieties of seeds, various environmental or climatic conditions of growing but also the different stages of seed maturation. It is worth noting that reliable estimation of phytate content is still flawed by inaccuracy and/or discrepancies resulting from inappropriate analytical tools used for the determination of InsP6 in whole food [31].

### 3.1. Reduced Supply from Food Sources

Since the 1970s, the concern for the (overestimated) antinutritional effect of phytic acid prompted the adoption of different strategies to counteract these unwarranted, negative consequences. Namely, studies have been designed to develop ways to eliminate phytate from foods by soaking and/or extracting the foods or by enhancing fermentation, thus creating phytate hydrolysis products that have weak mineral-binding properties. These measures may have likely reduced the InsP6/myo-Ins content of several aliments. It is, therefore, conceivable that low-vegetable/cereals consumers—especially in western countries—may have been exposed to a low-inositol diet, thus leading to a relative deficiency in the nutritional availability of inositols.

The myo-Ins assumption through the diet has rarely been investigated, and we have to rely on indirect estimations, based on the consumption of phytate-rich aliments. Moreover, systematic studies on the mean daily dietary phytic acid intake in humans are scarce. Nonetheless, available data evidence shows huge differences among different countries, and an even more relevant variability among different studies performed in the same country. At a first glance, the daily intake does not exceed 500–700 mg/day for western countries, while higher consumption data have been recorded in Africa and Asia. Yet, in both cases, a wide variability has been recorded, probably reflecting hidden diversities (of societal, educational, nutritional origins) among the population under observation. For example, in a study conducted in India, huge differences have been tracked depending on the age and gender of subjects [69]. Similar results have been reported among Egyptians [70] as well as in many other countries (reviewed in [31]). Moreover, in Europe, contrary to what is generally assumed (and uncritically reported in some studies), InsP6 intake is usually far below the supposed daily doses. For instance, in Italy, an early report has documented a broad range of 112–1367 mg phytic acid intake per day with an estimated mean value of 219 mg/day [71]. A further study stated the mean phytic acid intake of the national Italian diet approximates 293 mg, defined as the average of typical diets from the north-west (288 mg), from the northeast (320 mg), and from the south of Italy (265 mg) [72]. In both cases, the overall consumption of InsP6 is very low, though. Similar considerations apply for USA and Canada, where averaged InsP6 consumption values are far below the level usually indicated by many reports (i.e., ~1 g/day). A significant lower InsP6 intake has indeed recorded especially in younger people. Both US and Canadian children showed a mean daily phytate intake ranging from 170 to 390 mg/day [73,74], while in adults the median intake is significantly higher, averaging 538 mg/day, even if relevant differences have been found between Males and Females (608 mg vs. 512 mg/day, respectively) [75]. These data should be put in parallel with those obtained in adult Asian immigrants to Canada, consuming mostly a vegetarian diet, in which mean daily InsP6 intake steadily increases (1487 ± 791 mg/day) [76].

### 3.2. Impairment of Inositol Biosynthesis

Estimation of inositol deficit in mammals is further complicated by the fact that myo-Ins (as well as InsP6) can be synthetized by organisms. The difficulty lies on the fact that it is still a matter of research if inositol biosynthesis could compensate for deficiencies or increased functional requirements of inositol. An indirect appreciation about the relevance of inositol biosynthetic pathways can be drawn by considering that very high levels of myo-Ins are produced in the brain (reaching concentration up to 10–15 folds the plasma values), even if this tissue can only minimally “extract” myo-Ins from circulating blood [77].

Myo-Ins is synthetized from glucose-6-phosphate (G-6P) through two sequential biochemical reactions: G-6P is first isomerized by the NADH-dependent, cytosolic d-3-myoinositol-phosphate synthase (inositol synthase, INO1 or MIPS1, encoded by INO1 and Inositol-3-Phosphate Synthase 1, ISYNA1 gene in yeast and mammals respectively) to inositol-3-phosphate (Ins3P). Inositol synthase is expressed in all tissues and has the highest expression in testis, followed by heart, pancreas, ovary and placenta [78].

Inositol-3-phosphate is hence dephosphorylated by inositol monophosphatase-1 (IMPA-1 or IMPase) to yield free myo-Ins [79]. Free inositol may also be obtained by recycling inositol phosphates.

Living organisms—from yeast to mammals—can synthetize myo-Ins up to 4–5 g/daily. This estimation indicates that the overall myo-Ins daily requirement in basal conditions (including both external sources and endogenous synthesis), should not be below the threshold value of 5–6 g. Yet, as the biosynthetic capability hugely varies among different tissues and independent of changing functional requirements (morphogenesis, sperm production, etc.), it can be hypothesized that myo-Ins biosynthesis should change accordingly. Indeed, in yeast both INO1 and IMPA-1 are inducible upon inositol depletion [80], while being downregulated in presence of high inositol content [81,82]. Noticeably, conservation of function between yeast and humans has been demonstrated for MIPS, the rate-limiting enzyme in inositol synthesis [83].

Both INO1 and ISYNA1 activity can be modulated by epigenetic factors, eventually leading to alternatively spliced isoforms, one of which was shown to modulate enzyme activity negatively [84]. Additionally, ISYNA1 exhibits gender- and tissue-specific DNA methylation [85] that, overall, regulates the inositol biosynthetic pathway.

In yeast, INO1 transcription is under the control of the transcriptional repressor Opi1 in response to myo-Ins and phosphatidic acid (PA) levels [86]. Opi1 is stabilized by physically interacting with PA on the endoplasmic reticulum membrane. In the presence of myo-Ins deficit, PA levels increase concomitantly. Consequently, the association between Opi1 and the endoplasmic reticulum is further strengthened, and INO1 transcription is derepressed to increase inositol synthesis, probably involving also inositol pyrophosphates, which positively regulated INO1 transcription in yeast [87].

On the contrary, in mammal cells ISYNA1 activity is not regulated in response to intracellular myo-Ins availability [78]. Instead, ISYNA1 expression is mostly negatively modulated by inositol pyrophosphate, through inositol hexakisphosphate kinase (IP6K1), which catalyzes the formation of pyrophosphate (IP7) at position 5 of inositol pentakisphosphate/inositol hexakisphosphate [88]. Translocation of IP6K1 to the nucleus is facilitated by interaction with PA. In the nucleus, IP6K1 associates with chromatin and synthesizes IP7, which in turn inhibits transcription of ISYNA1 by increasing methylation of its sequences through histone modification [89].

Such a finding recognizes PA as a “metabolic sensor” that can indirectly modulate myo-Ins levels depending of the cellular energy requirements. It is noticeable that PA is synthetized from two glycolytic intermediates—dihydroxyacetone phosphate and glycerol 3-phosphate—and intracellular PA increases when glucose is metabolized at high rates, and cells necessitate sustained energy expenditure [90]. Moreover, high glucose levels indirectly increase the activity of phospholipase-D, a key enzyme in the PA synthesis [91], and decrease inositol synthase activity in rat testes [92].

Given that nuclear localization of IP6K1 is likely to increase in presence of high PA content (and consequently of inositol deficiency), this mechanism would paradoxically lead to a further deepening of myo-Ins deficit in cells already with low myo-Ins content. This negative feedback loop may eventually worsen the metabolic status of mammal cells, especially cancer and insulin-resistant cells that are tightly dependent on glucose metabolism [93]. In these conditions, the negative inhibition exerted by IP6K1 may probably induces a relative inositol-deficiency.

On the contrary, in mammalian cells, a positive regulator of ISYNA1 transcription has been identified in GSK3 (Glycogen synthase kinase 3). Some lines of evidence demonstrated that de novo synthesis of inositol is positively regulated by Mck1—a GSK3 homolog—which is required for normal activity of MIPS1, the enzyme catalyzing the rate-limiting step of inositol synthesis [94]. Indeed, GSK3 is required for optimal ISYNA1 activity, as loss of GSK3 activity causes myo-Ins depletion [95].

Finally, it is noticeable that ISYNA1 activity may be under hormonal control in the reproductive organs and liver of hypophysectomized and thyroidectomized rats, thus suggesting a “permissive” role exerted by thyroid and hypophysis-dependent hormones in the regulation of myo-Ins biosynthesis [96]. Noticeably, estrogens have been shown to stimulate inositol synthase in the uterus of ovariectomized female rats [97].

While in the past the inositol biosynthesis registered only limited interest, the above findings prompted a reconsideration of the critical physiological relevance of this pathway. Yet, despite the critical role myo-inositol and its phosphate metabolites plays in some organs (testes, brain), further studies are warranted to ascertain the importance of inositol biosynthesis in other tissues.

### 3.3. Reduced Absorption Due to Glucose-Dependent Inhibition of Inositol Uptake

A correlation among inositol, phytic acid and glucose metabolism was hypothesized long ago, when a negative correlation between phytate intake and glycemic index of cereal and legume foods consumed by humans was observed [98]. Indeed, removal of phytate from bean flour increased glycemic index compared to the whole bean flour [99]. Several lines of evidence have later shown that InsP6 was inhibiting amylase activity by complexing Ca ions, which are required for activating the degradation of complex carbohydrates [100]. In turn, myo-Ins significantly inhibits duodenal glucose absorption and reduced blood glucose increase, suggesting the existence of a competitive affinity for the transporter system [101].

The relationship between glucose and myo-Ins is an even more conflicting one, and several abnormalities in myo-Ins pathways has been associated with abnormalities of glucose metabolism [102]. Inositol is actively transported inside the cell through two different mechanisms, involving sodium ion-coupled and proton-coupled transporters (HMIT1), respectively [103]. The major transport system is represented by the Na-dependent system comprising two transporters—SMIT1 and SMIT2—that regulate myo-Ins uptake by brain and many other tissues [104]. Noticeably, myo-Ins transport ensured by SMIT1/2 increased after downregulation of protein kinase C (PKC) activity and decreased after activation of protein kinase-A in human cells, indicating that the inositol uptake system is post-translationally regulated through phosphorylation [105].

Sodium-based myo-Ins transporters are influenced in different ways by glucose, and glucose-dependent metabolic pathways. In hepatocytes, inhibitors of the SGLT1/2 glucose transporter system prevent inositol uptake. That finding clearly shows that myo-Ins shares with glucose some transporter systems [106]. Cellular inositol uptake is also decreased in the presence of high or even normal glucose concentrations. Namely, hyperglycemia induces myo-Ins depletion in nervous tissues, via competitive inhibition of sodium-dependent myo-Ins uptake [107]. By considering the structural similarities between myo-Ins and glucose, this result is far from surprising. Inositol uptake in cell cultures may be significantly inhibited by 20 mM glucose concentration [108]—a value currently used in laboratory investigations—and this finding is of utmost importance for properly modeling of in vitro experiments. In vitro, high ambient glucose attenuates myo-inositol concentrating capability via competitive inhibition. Noticeably, inhibition of aldose reductase displayed a trend toward near-normalization of myo-inositol accumulation in cultured cells, indicating that sorbitol affects the myo-Ins transport system [109].

Glucose may also induce myo-Ins depletion even through a biochemical pathway switching. Indeed, in diabetic patients, inositol depletion could arise due to the activation of the polyol pathway (glucose-sorbitol pathway), whereby glucose is first converted to sorbitol by aldose reductase and then to fructose by sorbitol dehydrogenase [110].

By exposing cells to high concentrations of glucose, intracellular myo-inositol content was reduced from 12.39 ± 0.64 nmol/mg protein at 0 mmol/L glucose to 6.54 ± 0.38 nmol/mg protein at 27.5 mmol/L glucose and 4.88 ± 0.43 nmol/mg protein at 55 mmol/L glucose. It is worth noting that the decrease of myo-inositol content was partially prevented by co-incubating cells with aldose reductase inhibitors [111].

Increased conversion of glucose into sorbitol significantly raises the intracellular osmolarity. To counteract that harmful event, cells actively inhibit the uptake of other relevant osmolytes (like inositol) and favor their cytosolic depletion, by downregulating the expression of specific carriers at the transcriptional level [112]. Indeed, pharmacological inhibition of aldose reductase restore myo-Ins content in impaired diabetic peripheral nerves [107], thus improving the sodium-potassium ATPase activity. Supplementation with myo-Ins can efficiently counteract such abnormalities, improving nerve conduction in diabetic animals [113,114].

Increase in extracellular glucose (within the range mimicking the one observed in diabetes) caused marked decrease in the synthesis of phosphatidylcholine, phosphatidylethanolamine, and phosphatidylinositol [115], resulting in chronic elevation of diacylglycerol (DAG), the chief physiological activator of protein kinase C (PKC) [116]. Moreover, the activation of the polyol pathway and the concomitantly reduction in cellular myo-Ins content can promote the oxidation of the NADPH/NADP+ and reduces the NADH/NAD+ redox couples, thereby perturbing a range of other adenine nucleotide-linked reactions. In diabetes, oxidation of NADPH/NADP+ may enhance susceptibility to oxidative tissue damage through depletion of reduced glutathione [117].

These findings not only provide further support to the hypothesis for which peripheral nerve integrity is highly dependent on the inositol-pathways [118], but also strengthen the link between hyperglycemia and deregulated inositol metabolism.

Glucose-dependent depletion of cellular inositol storage has received further confirmation by a compelling body of observations. Hyperglycemia has been associated with an intracellular myo-inositol depletion in some tissues, especially in those susceptible of developing diabetes complications [119]. Moreover, hyperglycemia and insulin-resistance modify the relative proportion—namely by modulating the myo-Ins/d-chiro-Ins ratio—in which different inositol isomers are represented in different tissues [120]. In experimentally induced diabetes, inositol depletion—resulting from both cellular depletion and increased inositol urinary excretion—is an early marker of hyperglycemia and insulin resistance [102].

Myo-inositol depletion, in turn, may worsen insulin resistance and diabetes complication, including disturbances in cellular redox and free radical defense, increased oxidative glycation stress [121,122], and renal function. Conversely, myo-Ins supplementation improves several diabetes symptoms, as well as metabolic markers in a wide range of pathological conditions [123,124].

### 3.4. Increased Renal Excretion and Catabolism

Inositol is chiefly catabolized in the kidney, where the enzyme myo-inositol oxygenase (MIOX) specifically metabolizes myo-Ins through the glucuronate-xylulose pathway. MIOX is exclusively expressed in the renal cortical tubules and is markedly downregulated in acute kidney injury [125]. MIOX catalyzes myo-Ins transformation into d-glucuronic acid, which is further metabolized into l-gulonate by aldehyde reductase. The latter is hence converted into xylulose and ribulose, which finally enter into the glycolytic pathway [126].

Nephrectomy in animals impairs myo-Ins degradation, while renal failure is associated with significant abnormalities in myo-Ins metabolism and both inositol plasma and urinary levels [127].

Furthermore, elevated inositol excretion (including both myo-Ins and d-Chiro-Ins) have been observed in diabetic animal models characterized by hyperglycemia and glycosuria, regardless of circulating insulin and body weight [128]. Previous studies on animal models of diabetes documented an increased urinary loss involving only one isomer (myo-Ins or d-Chiro-Ins) [129,130], or both [128]. Similarly, in humans, increased urinary myo-inositol has been consistently demonstrated in both type 2 diabetes [120,131,132], and type 1 diabetes [133]. On the contrary, d-chiro-Ins has been alternatively found increased [134,135] or reduced [133]. It is noticeable that, despite increased expression of the inositol transporter systems (SMT1/2), inositol reabsorption is significantly inhibited at the tubular level [136]. Other pathophysiological processes should therefore explain increased urinary loss of inositol isomers in diabetic kidneys [137].

Increased urinary excretion significantly contributes to depleting inositol, and may represent an independent relevant cause of inositol deficiency during both renal failure and diabetes. However, renal depletion of myo-Ins persisted despite normalization of sorbitol levels in diabetic rats treated with an aldose reductase inhibitor, strongly suggesting that inositol depletion occurs independently of the polyol pathway [138].

Indeed, it has recently been observed that myo-Ins depletion happens even in insulin-resistant or hypertensive animals, without involvement of the inositol biosynthetic capability, while MIOX activity steadily increases [139]. Furthermore, MIOX overexpression has been already observed in other models of animal diabetes [125,140]. It is argued that, at least in diabetic, hyperglycemic animals, MIOX overexpression may be fostered by xylose, an intermediate metabolite of the glucuronate-xylulose pathway (through a positive feedback), and further reinforced by xylulose-5-phosphate, which activates carbohydrate-responsive element binding protein [141]. In turn, it is worth noting that a sustained activation of the glucuronate-xylulose pathway would be a significant source of oxidative stress [126], thus contributing to fibrosis and progressive impairment of renal function [142]. Conversely, inhibition of MIOX expression reduces renal (tubular) damage, improving renal function in diabetic animals. MIOX overexpression is modulated by glucose-induced transcription factors such as NFAT-5, ChREBP, Nrf-2, AP1, and cAMP-response element-binding protein. Post-translational modifications, chiefly controlled by PKA, PDK1 and PKC, activate MIOX through phosphorylation of serine and threonine residues, mostly clustered in the N-terminal segment of the MIOX enzyme [143]. Aldehyde reductase is also strongly transcriptionally upregulated by high glucose ambience and oxidative stress stemming from the advanced glycation products [144]. Thereby, high glucose levels enhance myo-Ins catabolism by overexpressing and post-translationally activating both MIOX and aldehyde reductase. It is worth noting that the administration of antioxidants can strongly counteracts these effects, and improves the renal function [139].

These studies suggest a pivotal role of MIOX in diabetes-associated renal damage. Moreover, as MIOX overexpression is independent from the glucose-sorbitol pathway, that finding explain why by inhibiting aldose reductase (which catalyzes the main enzymatic step of that pathway) renal function could be only minimally improved in diabetic animals [138].

Diabetic nephropathy may directly arise from MIOX deregulation, as suggested by studies on renal mitochondria from diabetic animals. Indeed, both in vivo and in vitro investigations revealed that MIOX upregulation in the presence of high glucose levels induces mitochondrial fragmentation and depolarization, while inhibiting autophagic removal of damaged mitochondria through Pink1-dependent Mfn2–Parkin interaction [145]. The net effect of these events would be disruption in the surveillance of mitochondrial quality control, accumulation of dysfunctional organelles, generation of reactive oxygen species, and increased apoptosis, leading to tubular injury.

These findings suggest that deregulation of glucose metabolism will trigger abnormalities in myo-Ins metabolism and can increase the production of inositol-dependent toxic metabolites, which ultimately will damage renal function and promote inositol urinary loss. Therefore, it is likely that during hyperglycemia, insulin resistance and/or hypertension, changes in kidney metabolism are directly responsible for increased inositol loss, including both myo-Ins and d-Chiro-Ins isomers.

## 4. Hypothesis for Future Research

As already observed, it is remarkable that the incidence of some of the most diffused cancers (colon, breast) is superimposable to the distribution of the most relevant metabolic diseases (diabetes, obesity, and hypertension) [146]. For instance, diabetes and atherosclerosis, like the non-infective diseases of the bowel, have a comparable incidence in Afro-Americans and Caucasians in contrast to their rarity in rural Africa [147]. Furthermore, both these conditions, are relatively infrequent in Japan, but have increased in incidence in Japanese immigrated to the USA [148]. Overall, communities eating a western-like diet, rich in fat, sugar and significantly deprived of fibers share a relevant increased risk for both metabolic and cancerous diseases. This evidence raised has concerns since the 1970s, thus justifying issuing a warning against the removal of so much of the unabsorbable fiber from our food, and the associated over-ingestion of refined carbohydrates. More remarkable is that a low-fiber diet lacks some key components—such as phytates and inositols—for which a mechanistic link has been clearly established in the pathogenesis of both cancer and metabolic illness (diabetes and insulin resistance, PCOS, metabolic syndrome). Namely, depletion of cellular content of inositol (and of its isomers and phosphate derivatives), has been reported in both diabetic and cancerous tissues [149,150], while deregulation of myo-Ins metabolism has been found in a number of conditions (PCOS, metabolic syndrome), mechanistically and epidemiologically associated with high-glucose diet or altered glucose metabolism. Inositol deficit may first arise from low nutritional intake of phytate-rich foods, the principal alimentary source of myo-Ins. Given that currently available foods have been designed to eliminate phytate, it is tempting to speculate that dietary intake of InsP6 is significantly reduced. Indeed, several lines of evidence suggest that this is precisely the case.

**Figure 1:**
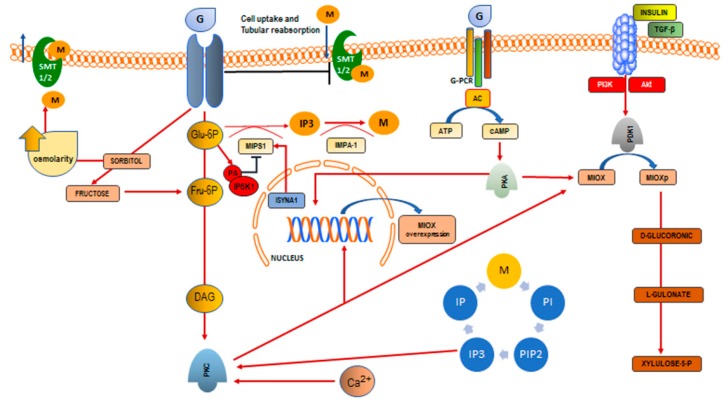
Glucose-inositol relationships. High glucose levels inhibit myo-Ins uptake by cells, by competing with SMT1/2 transporter systems. Moreover, through the activation of the polyol pathway, glucose extrudes myo-Ins from cells, thus “buffering” the increased osmolarity due to the augmented sorbitol levels. Biosynthesis of myo-Ins begins with G-6P enzymatic transformation into inositol-1-phosphate. However, high glucose levels indirectly inhibit myo-Ins biosynthesis, by increasing intracellular PA and consequently activating IP6K1, the principal negative regulator of myo-Ins de novo synthesis. Ultimately, glucose increases renal tubular catabolism and urinary loss of inositol through MIOX activation and overexpression. DAG, diacylglycerol; PKC, PKA, protein kinase C and A; G, glucose; G-6P, glucose-6-phosphate; Fru-6P, Fructose-6-phosphate; M, myo-inositol; IP6K1, inositol hexakisphosphate kinase; PA, phosphatidic acid; ISYNA1, d-3-myoinositol-phosphate synthase; IP3, inositol-3-phosphate; IMPA-1, inositol monophosphatase-1; MIOX, myo-inositol oxygenase; MIOXp, phosphorylated myo-inositol oxygenase; IP, inositol phosphate; PI, phosphatidyl-inositol; PIP2, phosphatidyl-inositol-4,5-biphosphate; PDK1, Pyruvate Dehydrogenase Kinase 1; AC, adenylate cyclase; G-PCR, G-protein coupled receptor.

Second, inositol deficiency may be “acquired”, downstream from the activation of several intracellular biochemical mechanisms (Figure 1).

It is truly amazing that glucose modulates, in some different ways, all of these pathways. High glucose content inhibits myo-Ins uptake by cells, the gut, and the kidney. Moreover, through the activation of the polyol pathway, glucose extrudes myo-Ins from cells, thus “buffering” the increased osmolarity due to the augmented sorbitol levels. Biosynthesis of myo-Ins begins with G-6P enzymatic transformation into inositol-1-phosphate. However, high glucose levels indirectly inhibit myo-Ins biosynthesis, probably by increasing intracellular PA and consequently activating IP6K1, the principal negative regulator of myo-Ins de novo synthesis. Ultimately, glucose increases catabolism and urinary loss of inositol through MIOX activation and overexpression. To sum up: high glucose levels hinder inositol availability by increasing its degradation and by inhibiting both myo-Ins biosynthesis and absorption. These underappreciated mechanisms may likely account for acquired, metabolic deficiency in inositol bioavailability. How these effects could participate in the pathogenesis of different degenerative diseases still necessitates further studies.

## Abbreviations

AC	adenylate cyclase
AP1	activator protein 1
ChREBP	carbohydrate responsive element-binding protein (ChREBP)
DAG	diacylglycerol
d-Chiro-Ins	d-Chiro-Inositol
G-6P	glucose-6-phosphate
G-PCR	G-protein coupled receptor
GSK3	glycogen synthase kinase 3
HMIT1	H^+^/myoinositiol co-transporter
IMPA-1/IMPase	inositol monophosphatase-1
INO1	Inositol-3-phosphate synthase
Ins3P/IP3	inositol-3-phosphate
InsP6	Inositol-hexakisphosphate or phytic acid
IP	inositol phosphate
IP6K1	inositol hexakisphosphate kinase
IP7	pyrophosphate
ISYNA1	Inositol-3-Phosphate Synthase 1
Mfn2	Mitofusin-2
MIOX	myo-inositol oxygenase
MIPS1	myo-inositol-1-phosphate synthase 1
myo-Ins	myo-Inositol
NFAT-5	nuclear factor of activated T-cells 5
Nrf-2	nuclear factor (erythroid-derived 2)-like 2
PA	phosphatidic acid
PCOS	polycystic ovary syndrome
PDK1	pyruvate dehydrogenase kinase 1
PI	phosphatidylinositol
Pink 1	PTEN-induced putative kinase 1
PIP2	phosphatidyl-inositol-4,5-biphosphate
PKA	protein kinase A
PKC	protein kinase C
SGLT1/2	sodium glucose transporter
SMIT1/2	sodium/inositol symporter
